# Analysis of mRNA and miRNA expression in RAW 264.7 macrophages infected with *Salmonella enterica subsp. enterica* serovar Dublin in the early stage of infection

**DOI:** 10.3389/fvets.2026.1794290

**Published:** 2026-05-04

**Authors:** Zi Wang, Miao Sun, Zipeng Zhang, Yongqiang Wang, Shengjie Tong, Linghao Meng, Dongxu Han, Xinran Ge, Hengli Chen, Dahan Yang, Kai Liu

**Affiliations:** 1 College of Animal Science and Technology, Inner Mongolia Minzu University, Tongliao, China; 2 Inner Mongolia Engineering Technology Research Center for Prevention and Control of Beef Cattle Diseases, Tongliao, China; 3 Beef Cattle Industry School of Inner Mongolia Autonomous Region, Tongliao, China; 4 Zhalantun Vocational College, Hulunbeier, China; 5 Tongliao Agriculture and Animal Husbandry Development Center, Tongliao, China

**Keywords:** apoptosis, immune response, macrophage, miRNA, mRNA, *Salmonella enterica subsp. enterica* serovar Dublin

## Abstract

*Salmonella enterica subsp. enterica* serovar Dublin (*Salmonella* Dublin) is a host-adapted pathogen that causes severe systemic disease in cattle and presents a zoonotic risk to humans. While macrophages play a pivotal role in host defense against *Salmonella* they are also an intracellular niche for bacterial persistence. However, the mechanism underlying the early transcriptional response of macrophages to *Salmonella* Dublin infection is poorly understood. This study used high-throughput sequencing to profile mRNA and miRNA expression in RAW 264.7 mouse macrophages 2 h after infection with *Salmonella* Dublin compared to uninfected control cells. A total of 1,080 differentially expressed (DE) mRNAs were identified, of which 492 were up-regulated and 588 were down-regulated. In addition, 23 DE miRNAs, 18 up-regulated and 5 down-regulated, were identified. Meanwhile, KEGG enrichment analysis indicated significant enrichment of the DE mRNAs and miRNAs in signaling pathways associated with macrophage immune activation, including the TNF, HIF-1, and MAPK signaling pathways, as well as those involved in responses to *Salmonella* infection. The accuracy of the mRNA sequencing results was verified using RT-qPCR. In conclusion, the findings showed significant up-regulation of *Acod1*, *Ccl22*, *Bcl2a1b*, *miR-146-3p*, and *miR-150-5p in* the early stages of *Salmonella* Dublin infection of mouse macrophages, suggesting the involvement of these molecules in regulating the host immune response.

## 1 Introduction

*Salmonella* is a Gram-negative bacterium belonging to the *Enterobacteriaceae* family and commonly found in animal intestines. The bacteria are rod-shaped and motile, and facultative anaerobes (1, 2). *Salmonella* infects a wide variety of animal species, including humans, livestock, and poultry. The genus comprises approximately 2,600 serotypes, including *Salmonella enterica subsp. enterica* serovar Dublin (*Salmonella* Dublin), a serotype that is highly adapted to cattle but can also infect other animals including humans (3–6). *Salmonella* Dublin, unlike most other *Salmonella* serovars, tends to cause systemic disease, primarily manifesting as septicemia and respiratory disease in younger animals and reproductive disorders in older animals, with outbreaks associated with high levels of morbidity, mortality, and economic loss (7–10). *Salmonella* Dublin is most frequently reported serotype in European cattle, and can cause respiratory disease in calves between 10 and 30 days old, with an incidence rate of over 20% and mortality of 75% (11). In Germany, *Salmonella* Dublin caused 30%–45% of all Salmonellosis outbreaks in cattle herds in the years 2017–2019 (12). *Salmonella* Dublin is found globally where it results in serious economic losses, adversely affecting livestock husbandry and breeding (13). *Salmonella* Dublin has also received attention as it represents a zoonotic hazard (14).

Macrophages represent the first line of defense against pathogens, linking the innate and adaptive immune systems. Macrophages have multiple functions, including pathogen recognition, phagocytosis, microbial killing, cytokine secretion, stimulation of adaptive immunity, and production of superoxide radicals and hydrogen peroxide (15, 16). Accumulated evidence indicates that several intracellular pathogens, including *Salmonella*, can manipulate macrophage polarization to evade host immune defenses and prolong their survival in the host (17). Macrophages are considered a favorite niche for *Salmonella* to divide and persist within the host organism. Following infection, *Salmonella* may survive passage through the stomach to reach the intestine, where it can cross the intestinal barrier and subsequently be internalized by phagocytes such as macrophages (18). Within the macrophage, *Salmonella* secretes effector proteins to produce a specific *Salmonella*-containing vacuole (SCV) that can evade phagocytic cell killing (19). The pathogen can replicate within the SCV, leading to the formation of persister cells and reinvasion of epithelial cells or other phagocytic cells of the immune system (20). Thus, as macrophages represent the preferred *Salmonella* niche, the bactericidal activity of these cells plays an important role in clearance of the pathogen, with many of these adaptive processes regulated at the transcriptional level (21).

Small RNAs, such as microRNAs (miRNAs), have emerged as essential regulators of macrophage function. miRNAs are a class of 17 to 25-nt small noncoding RNAs (ncRNAs) (22). Unlike mRNAs, miRNAs do not code for protein but bind to the 3′ untranslated regions (3′ UTRs) or open reading frames (ORFs) of the target mRNA, resulting in translational repression or mRNA degradation, thereby regulating post-transcriptional gene expression (23–25). MiRNAs have been confirmed to play key roles in many biological processes, including the cell cycle, differentiation, apoptosis, and pathogenesis (26). Recent evidence has demonstrated the importance of miRNAs in regulating innate immune responses induced by bacterial, viral, fungal, and protozoan infections, and that miRNA expression patterns are negatively correlated with the expression of their targets (25, 26). In addition, a study found that *Salmonella* activates NF-κB in mouse macrophages, thereby modulating the expression of various miRNAs, including *miR-21*, *miR-146* a, and *miR-155* (27). It has also been observed that these miRNAs are significantly up-regulated in rat mononuclear cells after *Salmonella* infection and regulate the proliferation of T and B cells (26, 28). In addition, down-regulation of *Let-7* miRNA family members during *Salmonella* infection induced the release of cytokines IL-6 and IL-10, which are involved in immune responses to *Salmonella* infection in macrophages (26).

High-throughput sequencing is an effective method to reveal the function and molecular composition of cells and tissues, and has contributed significantly to the understanding of development and disease in various organisms. Our previous research demonstrated regional circulation of *Salmonella* Dublin, and successfully isolated a *Salmonella* Dublin strain of bovine origin (29). However, relatively little is known of macrophage involvement during the early stages of infection. Therefore, this study aimed to investigate changes in the mRNA and miRNA expression profiles and their associated immune regulatory mechanisms in macrophages during the early stage (2 h) of *Salmonella* Dublin infection. *Salmonella* Dublin was co-cultured with RAW 264.7 mouse macrophages, and transcriptome sequencing was used to identify differentially expressed (DE) mRNAs and miRNAs, enabling the construction of regulatory networks based on these DE mRNAs and miRNAs to elucidate their interactions during the early stage of infection.

## 2 Materials and methods

### 2.1 Cells and cell culture

Mouse monocyte–macrophage RAW 264.7 cells were purchased from Shanghai Anwei Biotechnology Co., Ltd. (Shanghai, China) and were grown in Dulbecco’s modified Eagle medium (DMEM) supplemented with 10% fetal bovine serum (FBS, Hyclone, Logan, UT, USA) and 1% penicillin and streptomycin at 37 °C with 5% CO_2_.

### 2.2 Bacterial culture and infection

The *Salmonella* Dublin was isolated from a large -scale beef cattle farm in Tongliao City and kept in the Laboratory of Preventive Veterinary Medicine, College of Animal Science and Technology, Inner Mongolia MINZU University (29). The *Salmonella* Dublin strains were routinely cultured overnight in brain heart infusion (BHI) broth at 37 °C with shaking at 200 rpm. Bacterial growth was monitored by measuring absorbances at OD600. Bacteria in the logarithmic growth phase(OD600 = 0.6) were collected, resuspended in RPMI 1640 medium, washed, and resuspended to prevent the formation of bacterial clumps. The concentration was adjusted to 1 × 10^7^ CFU/mL for subsequent macrophage infection. RAW 264.7 cells were divided into experimental and control groups, with three biological replicates per group. The experimental group was infected with *Salmonella* Dublin strain at a multiplicity of infection (MOI) of 10:1. After 2 h, post-infection, the medium was replaced with fresh culture medium containing gentamicin (100 μg/mL) to eliminate extracellular bacteria. This was validated in preliminary experiments by plating the culture supernatant on BHI agar, which yielded no bacterial colonies, confirming the efficacy of this concentration in clearing extracellular *Salmonella* Dublin. The control group remained uninfected. Both groups were re-cultured at 37 °C and 5% CO_2_ for 2 h and then collected for RNA extraction (30).

### 2.3 Library preparation, sequencing and data analysis

RNA was extracted using TRIzol reagent (Invitrogen, Waltham, MA, USA) and treated with RNase-free DNase I (Takara, Kusatsu, Japan). The RNA was quantified using a NanoDrop spectrophotometer (Thermo Fisher Scientific, Waltham, MA, USA), and the quality and integrity were assessed using an Agilent 2,100 Bioanalyzer (Agilent Technologies, Santa Clara, CA, USA). Sequencing libraries were generated using a NEBNext Ultra RNA Library Prep Kit for Illumina (NEB, Ipswich, MA, USA), as directed, with the addition of index codes added for sample identification. For small RNA libraries, the NEBNext Multiplex Small RNA Library Prep Set for Illumina (NEB, USA) was used. After adapter ligation and PCR amplification, products were separated on an 8% polyacrylamide gel (100 V, 80 min), and fragments of 140–160 bp (small RNA plus adapters) were excised and recovered. Library quality was assessed on the Agilent Bioanalyzer 2,100 system. The libraries were sequenced on an Illumina Novaseq 6,000 platform by the Beijing Allwegene Technology Company Ltd. (Beijing, China), generating paired-end 150-bp reads (for transcriptome) and 50-bp single-end reads (for small RNA). To obtain high-quality clean reads, reads containing adapters and poly-N, as well as low-quality reads, were removed from the raw reads. At the same time, the Q20, Q30, GC contents, and sequence duplication levels of the clean data were calculated. After data processing, the clean reads were mapped to the reference genome sequence (*Mus musculus*, strain C57BL/6 J, RefSeq assembly GCF_000001635.27, GRCm39) using STAR, with annotation and further analysis based on the reference genome.

### 2.4 Functional enrichment of DE mRNAs and miRNAs

Differential expression of mRNAs and miRNAs was examined using the DESeq R package (1.10.1). The *p*-values were adjusted using the Benjamini and Hochberg method and a corrected *p*-value of 0.05 was used as the threshold for differential expression. The GOseq R package and KOBAS software were used for analyzing Gene Ontology (GO) functional terms and Kyoto Encyclopedia of Genes and Genomes (KEGG) pathways, respectively, in which the differentially expressed genes (DEGs) were involved (31–33).

### 2.5 Construction of the miRNA-mRNA correlation network

Interactions between miRNAs and mRNAs were analyzed using the miRNA and transcriptomic data, with the construction of an miRNA-mRNA interaction network using Cytoscape v3.10.4 software.^1^ The network was visualized using the built-in yFiles Layout tool, and edges were defined based on inverse expression patterns with |log2(fold change)| ≥ 1 for both miRNAs and mRNAs.

### 2.6 Real-time quantitative PCR (RT-qPCR)

To verify and characterize the identified DE miRNAs and mRNAs, RT-qPCR was used to measure the expression of 22 DE miRNAs and mRNAs. For miRNA RT-qPCR, stem-loop reverse transcription was used. RT-qPCR was performed using a SYBR Green PCR Master Mix Reagent Kit (TaKaRa, Japan), with *β*-actin and U6 genes used as internal references (34). Each 20 μL reaction contained 10 μL of 2 × SYBR Green Master Mix, 0.4 μL of each forward and reverse primer (10 μM), 1 μL of template, and 8.2 μL of ddH_2_O. The thermal cycling protocol was as follows: initial denaturation at 95 °C for 30 s, followed by 40 cycles of 95 °C for 10 s and 60 °C for 30 s, concluding with a melt curve analysis stage. Relative expression levels were calculated using the 2^−∆∆Ct^ method. The RT-qPCR primers are listed in Supplementary Table S1. Three independent replicates were used for each assay.

### 2.7 Statistical analysis

Statistical analyses were performed using GraphPad Prism 5 (GraphPad Software, San Diego, CA, USA). One-way analysis of variance (ANOVA) was used for multiple comparisons. All data are presented as mean ± standard error of the mean derived from three independent experiments, with a *p*-value of less than 0.05 considered statistically significant.

## 3 Results

### 3.1 Sequencing statistics, quality control, and comparison with the reference genome

The transcriptomes of *Salmonella* Dublin*-*infected and non-infected RAW 264.7 cells were sequenced. The average numbers of total reads from the three libraries obtained from the experimental and control groups were 42,889,754 and 41,798,664, respectively. Removal of Q ≤ 20 sequences, adaptor sequences, and sequences containing more than 10% of base information that could not be determined (N), resulted in 42,362,608 and 41,321,371 from the two groups. The original reads, valid reads, Q20, Q30, and GC contents of the two samples are given in Table 1. The Q20 and Q30 values were all above 90%, and the GC contents were normal. Bowtie software was used to compare the clean data with the reference genome. Information on the mapping of reads to the reference genome is shown in Table 2. miRNAs of 21 nt in length were the most abundant species in the control group, while those with lengths of 22 nt were the most abundant in the experimental group.

**Table 1:** Sequencing information on the different samples.

Sample name	Raw reads	Valid reads	Q20	Q30	GC content
Control group	42,124,360	41,709,622	96.80%	91.56%	49.88%
Control group	41,489,394	40,916,820	96.63%	91.21%	49.81%
Control group	41,782,236	41,337,670	96.62%	91.17%	49.80%
Experimental group	42,482,828	41,935,962	96.58%	91.12%	49.76%
Experimental group	41,347,964	40,899,796	96.53%	91.01%	49.86%
Experimental group	44,838,470	44,282,066	96.71%	91.39%	49.86%

**Table 2:** Information on sequencing and mapping of data to the reference genome.

Sample name	Total reads	Total mapped	Multiple mapped	Uniquely mapped
Control group	41,709,622	39,068,058 (93.67%)	2,462,578 (5.9%)	36,605,480 (87.76%)
Control group	40,916,820	38,056,082 (93.01%)	2,316,168 (5.66%)	35,739,914 (87.35%)
Control group	41,337,670	38,106,058 (92.18%)	2,330,600 (5.64%)	35,775,458 (86.54%)
Experimental group	41,935,962	38,915,996 (92.8%)	2,415,724 (5.76%)	36,500,272 (87.04%)
Experimental group	40,899,796	37,999,146 (92.91%)	2,309,772 (5.65%)	35,689,374 (87.26%)
Experimental group	44,252,066	41,111,850 (92.9%)	2,526,444 (5.71%)	38,585,406 (87.19%)

### 3.2 Analysis of DE mRNAs

**Figure 1:**
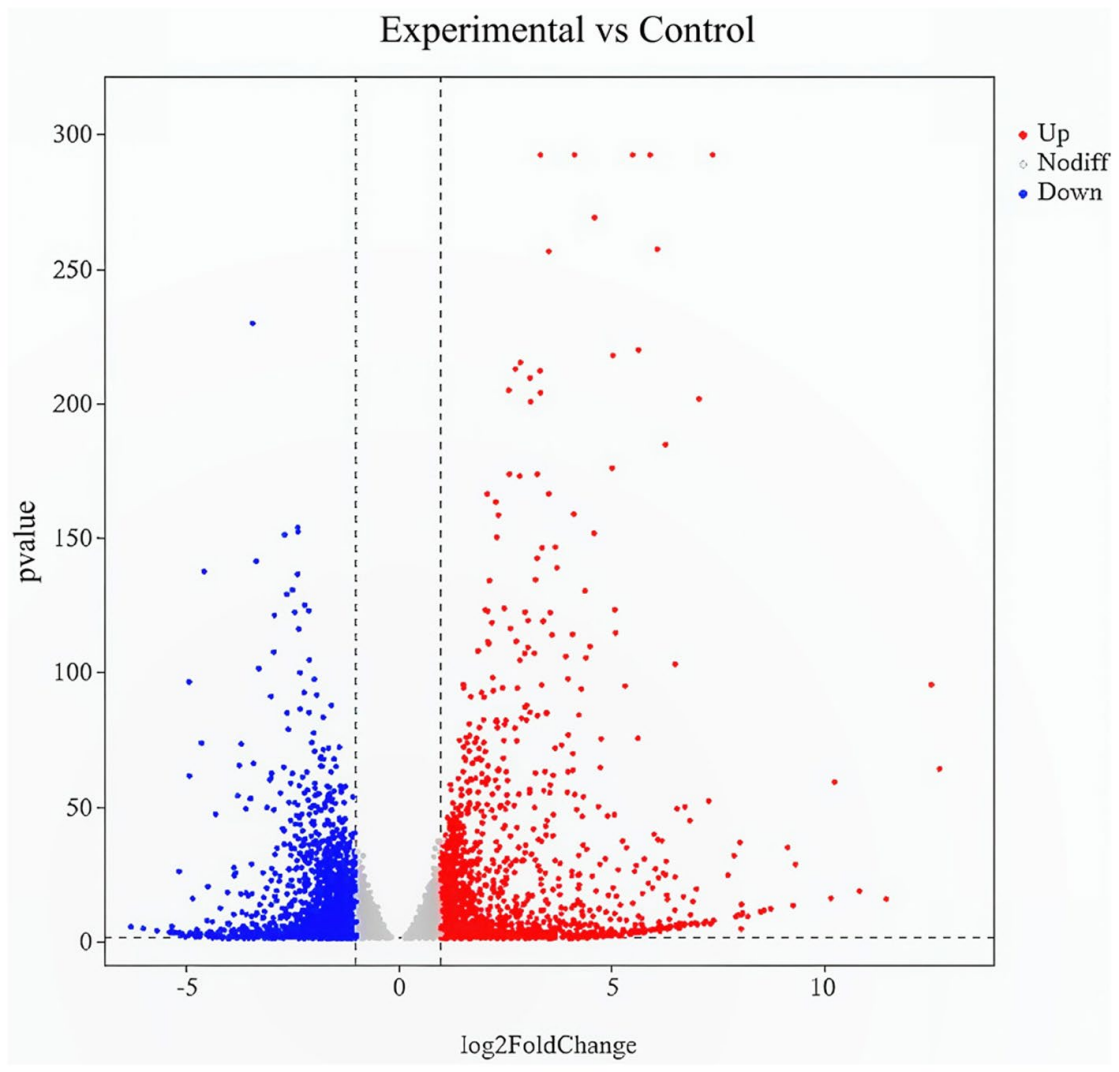
Volcano plot of DE mRNAs. A scatter plot shows the correlation of gene abundance. Red, blue, and gray dots represent up-regulated, down-regulated, and unchanged genes, respectively.

**Figure 2:**
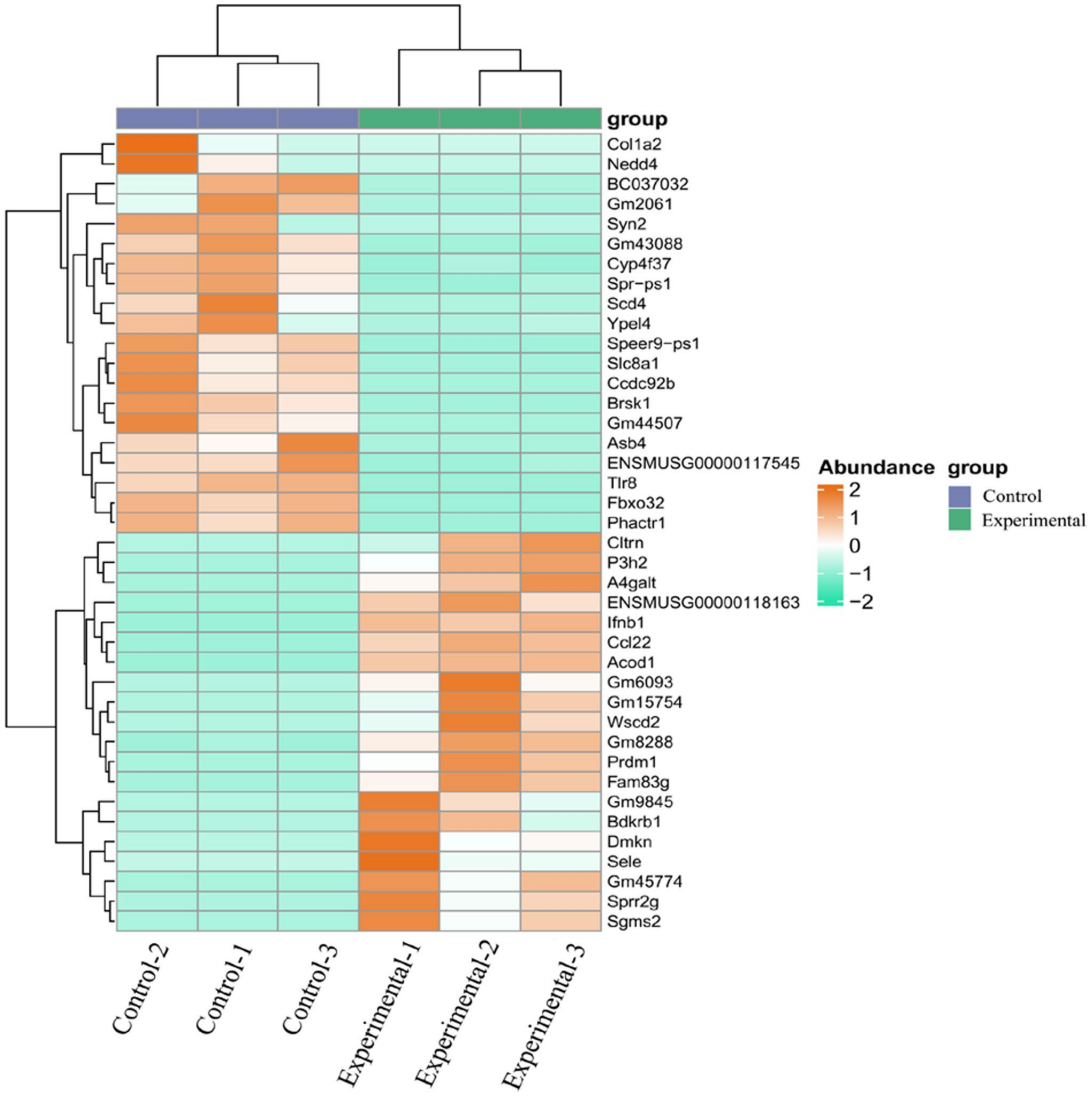
Cluster analysis of DE mRNAs. Samples are shown on the abscissa and mRNAs on the ordinate. Orange indicates high expression and green low expression.

DE mRNAs were identified using the criteria of |log2 (FoldChange)| > 1 and *p*-value (*p*val) < 0.05. A total of 1,080 DE mRNAs were identified, of which 492 were up-regulated and 588 were down-regulated. The most significantly up-regulated DEGs were *Ccl22*, *Sele*, *Acod1*, *Sgms2*, *Bdkrb1*, *Fam83g*, *Wscd2*, *GM15754*, *P3h2*, and *Gm6093*, while *Speer9 - ps1*, *Ccdc92b*, *Fbxo32*, *Gm44507*, *Gm43088*, *Slc8al*, *Tlr8*, *Brsk1*, *Asb4,* and *Colla2* were the most significantly down-regulated genes (Table 3). Volcano plots (Figure 1) and heatmaps (Figure 2) were used to visualize the DE mRNAs.

**Table 3:** The top 20 up-regulated and down-regulated DE mRNAs (*p*-value ≤ 0.05) for experimental vs. control macrophage samples as ranked by fold change.

Gene name	Description	Log2 (fold change)	*p*-value
*Ccl22*	Chemokine (C–C motif) ligand 22	7.3901	0
*Sele*	Selectin, endothelial cell	6.1539	0.00013522
*Acod1*	Aconitate decarboxylase 1	5.9172	0
*Sgms2*	Sphingomyelin synthase 2	5.816	0.00014384
*Bdkrb1*	Bradykinin receptor, beta 1	5.7884	0.00032123
*Fam83g*	Family with sequence similarity 83, member G	5.7256	0.00013812
*Wscd2*	WSC domain containing 2	5.7204	0.00026332
*Gm15754*	Predicted gene 15,754	5.7169	0.00024373
*P3h2*	Prolyl 3-hydroxylase 2	5.7107	0.00016966
*Gm6093*	Predicted gene 6,093	5.6832	0.00026913
*Speer9-ps1*	Spermatogenesis associated glutamate (E)-rich protein 9, pseudogene 1	−5.6778	0.00010132
*Ccdc92b*	Coiled-coil domain containing 92B	−5.3782	0.00045686
*Fbxo32*	F-box protein 32	−5.3214	0.00044969
*Gm44507*	Predicted gene 44,507	−5.3203	0.00063522
*Gm43088*	Predicted gene 43,088	−5.2585	0.00066068
*Slc8a1*	Solute carrier family 8 (sodium/calcium exchanger), member 1	−5.2019	0.00091199
*Tlr8*	Toll-like receptor 8	−5.1355	0.00098219
*Brsk1*	BR serine/threonine kinase 1	−5.1353	0.0011177
*Asb4*	Ankyrin repeat and SOCS box-containing 4	−5.0727	0.0017153
*Col1a2*	Collagen, type I, alpha 2	−5.0004	0.018779

### 3.3 GO and KEGG enrichment analyses of DE mRNAs

**Figure 3:**
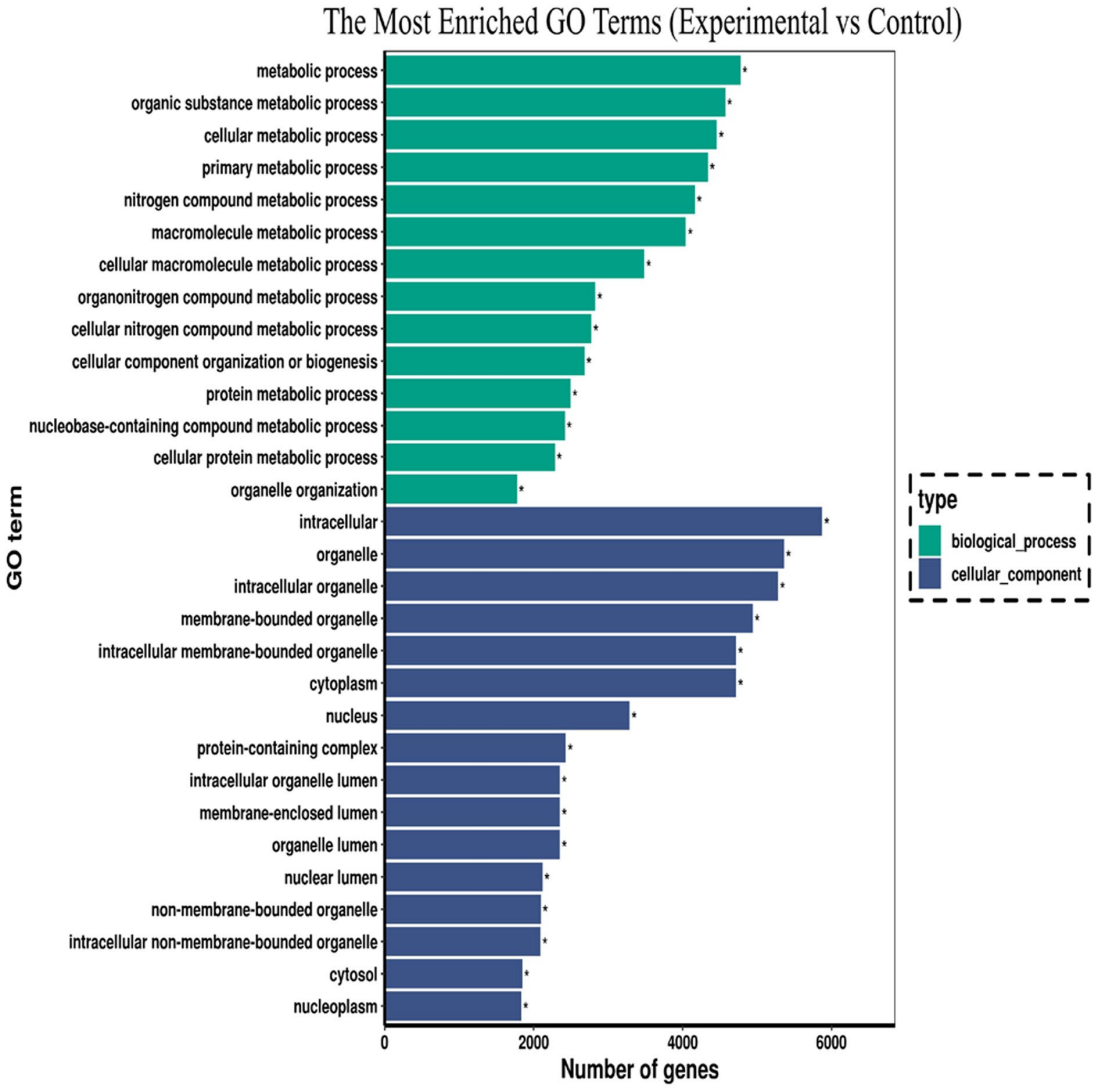
GO enrichment of DE mRNAs.

**Figure 4:**
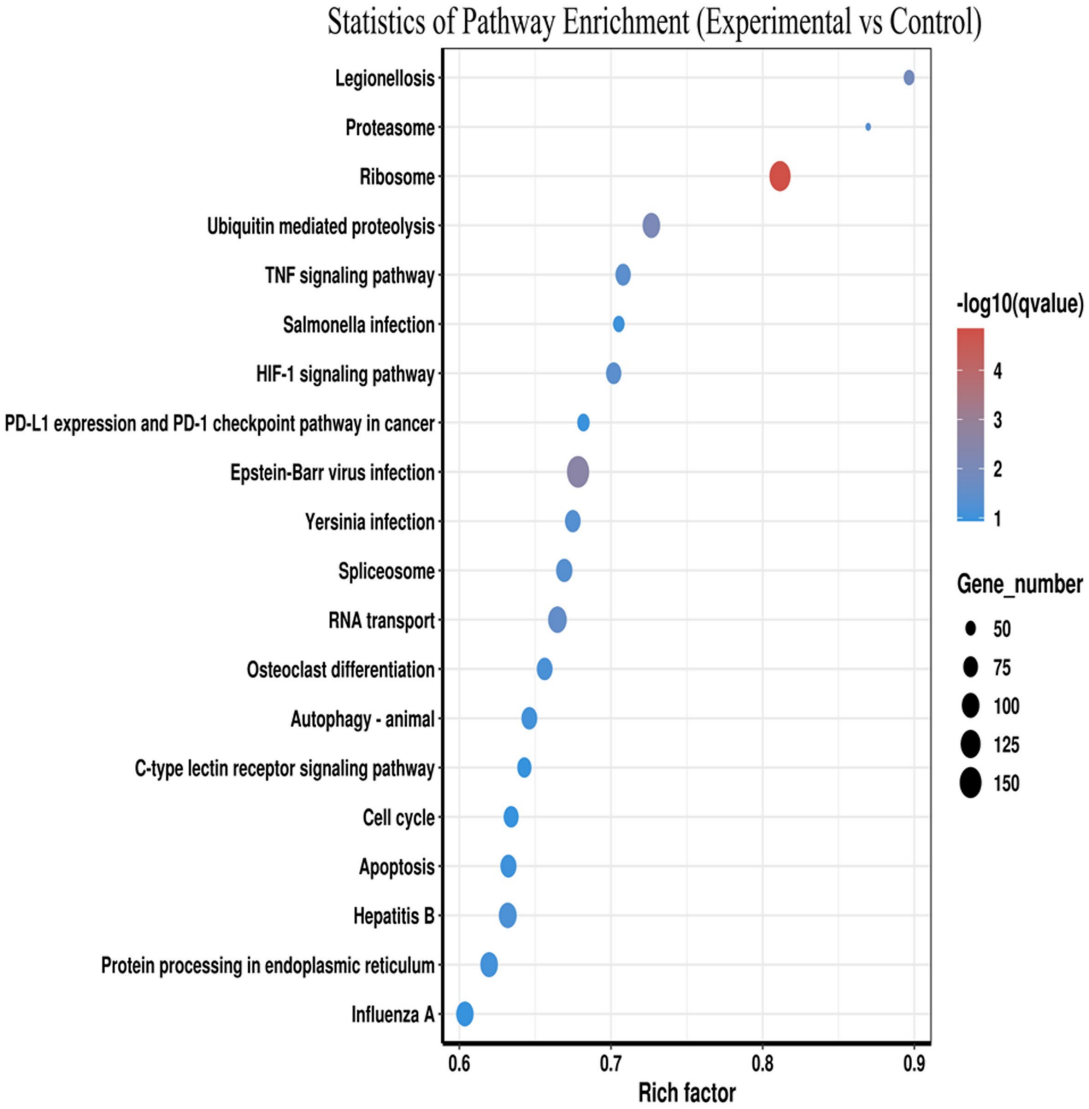
KEGG enrichment of DE mRNAs. The abscissa rich factor indicates the number of DE mRNAs. Pathways are shown on the ordinate.

The potential biological functions of the DE mRNAs were investigated using GO and KEGG enrichment analyses. GO functional annotation results showed that DE mRNAs was mainly enriched in cellular component and biological process, but no enrichment was found in molecular functional categories. In the biological process category, metabolic processes (GO: 0008152) were the most significantly enriched, followed by organic substance metabolic processes (GO: 0071704). In terms of cellular components. “intracellular” (GO: 0005622) was the most significant, followed by “organelles” (GO: 0043266). The top 30 GO terms with the most significant enrichment were included in the GO annotation map (Figure 3). The KEGG enrichment analysis revealed that the DE mRNAs were enriched in a total of 324 pathways, among which multiple pathways closely related to immune regulation, metabolism, and cell survival were significantly enriched following *Salmonella* infection of macrophages. These included the TNF signaling pathway, apoptosis, HIF-1 signaling pathway, C-type lectin receptor signaling pathway, and *Salmonella* infection, suggesting that these pathways play critical roles in the defense of macrophages against intracellular *Salmonella* infection. According to the enriched Q-value, 20 pathways with the most significant enrichment were selected and the results were displayed in the form of scatter plots (Figure 4). Further analysis of the genes enriched in the *Salmonella* infection pathway revealed a large number of upregulated genes involved in cytoskeletal remodeling, pro-inflammatory responses, and chemokine secretion, such as *Actb*, *Ccl3*, *Ccl22*, and *Il6*. This indicates that in the early stage of infection, macrophages may enhance the expression of chemokines to recruit adaptive immune cells for subsequent immune regulation and tissue repair, while also limiting the spread of the pathogen by enhancing the pro-inflammatory response. In addition, multiple key genes related to the regulation of inflammation and cell survival were found to be enriched in the TNF signaling pathway and the apoptosis pathway, suggesting that when facing *Salmonella* infection, macrophages may sustain cell survival by upregulating anti-apoptotic molecules (such as *Bcl2a1b*), thereby ensuring the continuous execution of immune functions.

### 3.4 Analysis of DE miRNAs

**Figure 5:**
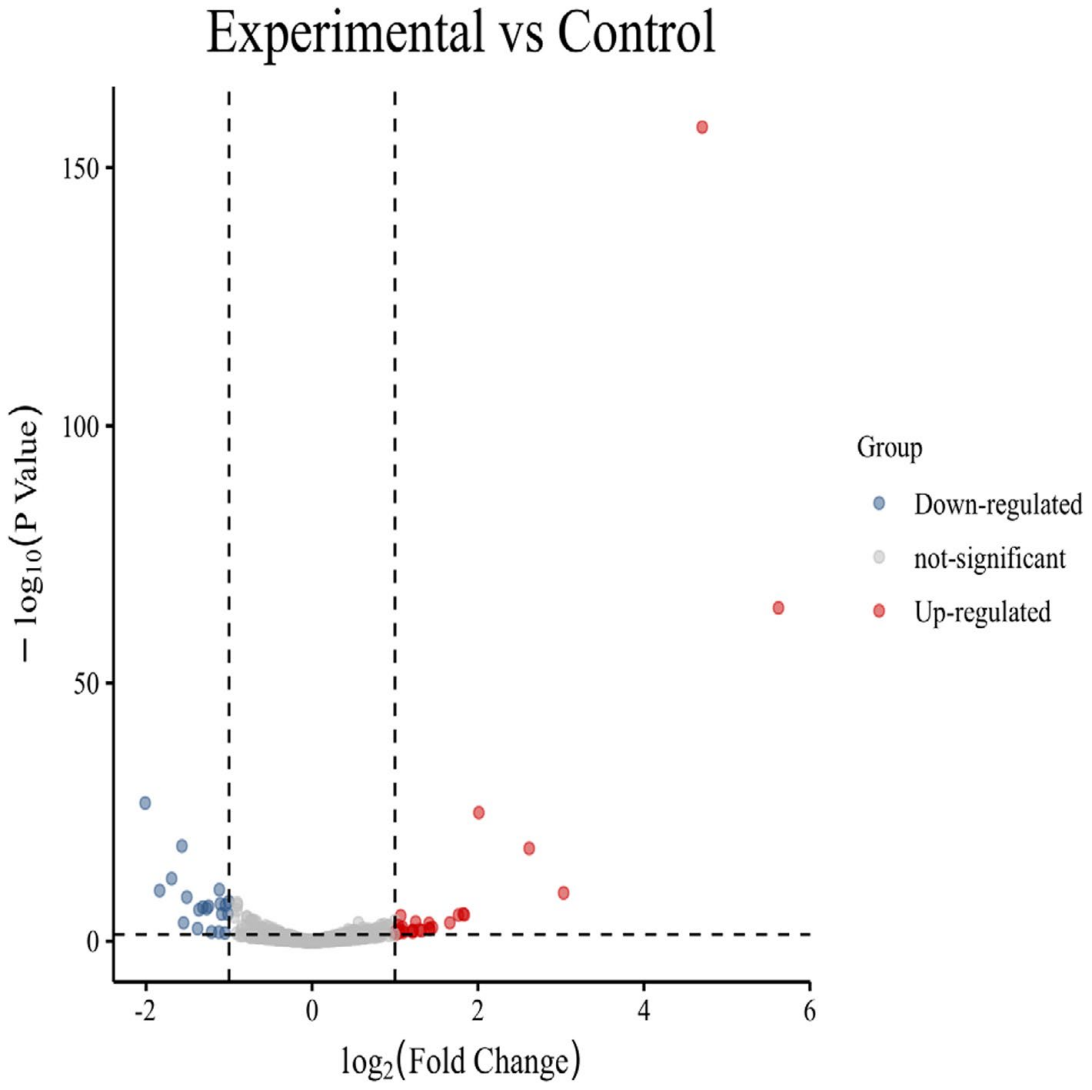
Volcanic plot of DE miRNAs. The scatter plot indicates abundance. Red, blue, and gray dots indicate up-regulation, down-regulation, and unchanged, respectively.

**Figure 6:**
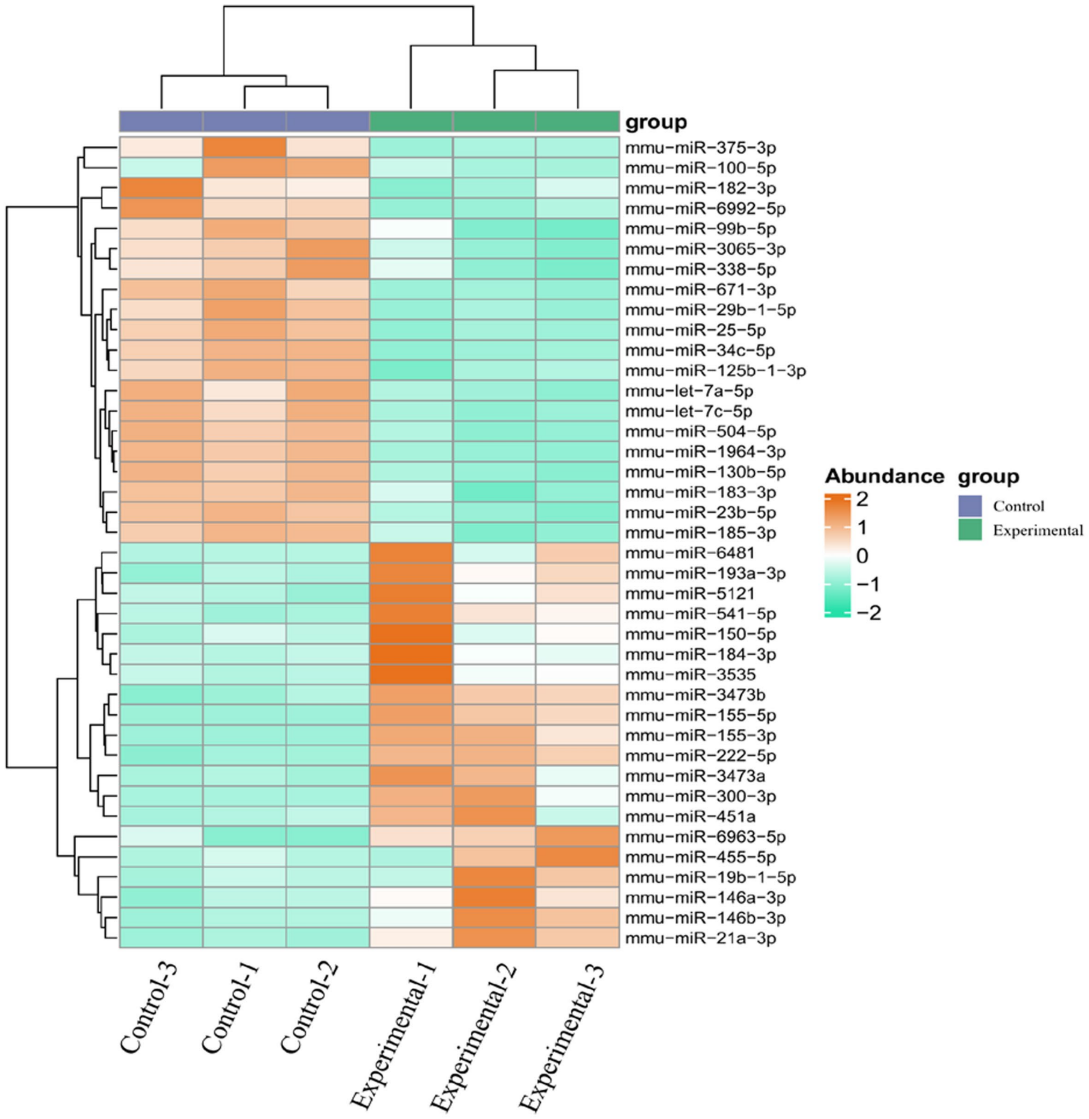
Cluster analysis of DE miRNAs. Samples are shown on the abscissa and miRNAs on the ordinate. Orange indicates high expression and green represents low expression.

Using the DESeq R package to identify DE miRNAs between two groups. Among them, 23 DE miRNAs had a *p*-value < 0.05 and |log2(fold change)| > 1, including 18 up-regulated and 5 down-regulated miRNAs (Table 4). The DE miRNAs are illustrated in a volcano plot (Figure 5) and a heatmap (Figure 6).

**Table 4:** Up-regulated and down-regulated DE miRNAs with significant differences (*p*-value ≤ 0.05) between experimental vs. control macrophage samples.

Gene name	Log2 (fold change)	*p*-value
*miR-146a-3p*	1.6609	0.00026786
*miR-3473a*	1.4513	0.0023236
*miR-6963-5p*	1.4156	0.0050387
*miR-3535*	1.4103	0.00032157
*miR-541-5p*	1.3198	0.0088988
*miR-184-3p*	1.2931	0.0063271
*miR-3473b*	1.2492	0.00017603
*miR-455-5p*	1.2173	0.0081693
*miR-150-5p*	1.213	0.017166
*miR-19b-1-5p*	1.1027	0.017373
*miR-146b-3p*	1.0894	0.0020978
*miR-1938*	1.0822	0.013952
*miR-744-3p*	1.0551	0.017718
*miR-1291*	1.0526	0.0052312
*miR-92a-1-5p*	1.044	0.00097729
*miR-129-1-3p*	1.0135	0.041874
*miR-122-5p*	1.0007	0.02147
*miR-122b-3p*	1.0007	0.02147
*miR-29b-1-5p*	−1.5484	0.00027312
*miR-6992-5p*	−1.3796	0.003811
*miR-375-3p*	−1.2111	0.017316
*miR-183-3p*	−1.1218	0.018017
*miR-182-3p*	−1.0492	0.02688

### 3.5 GO and KEGG enrichment of DE miRNAs

**Figure 7:**
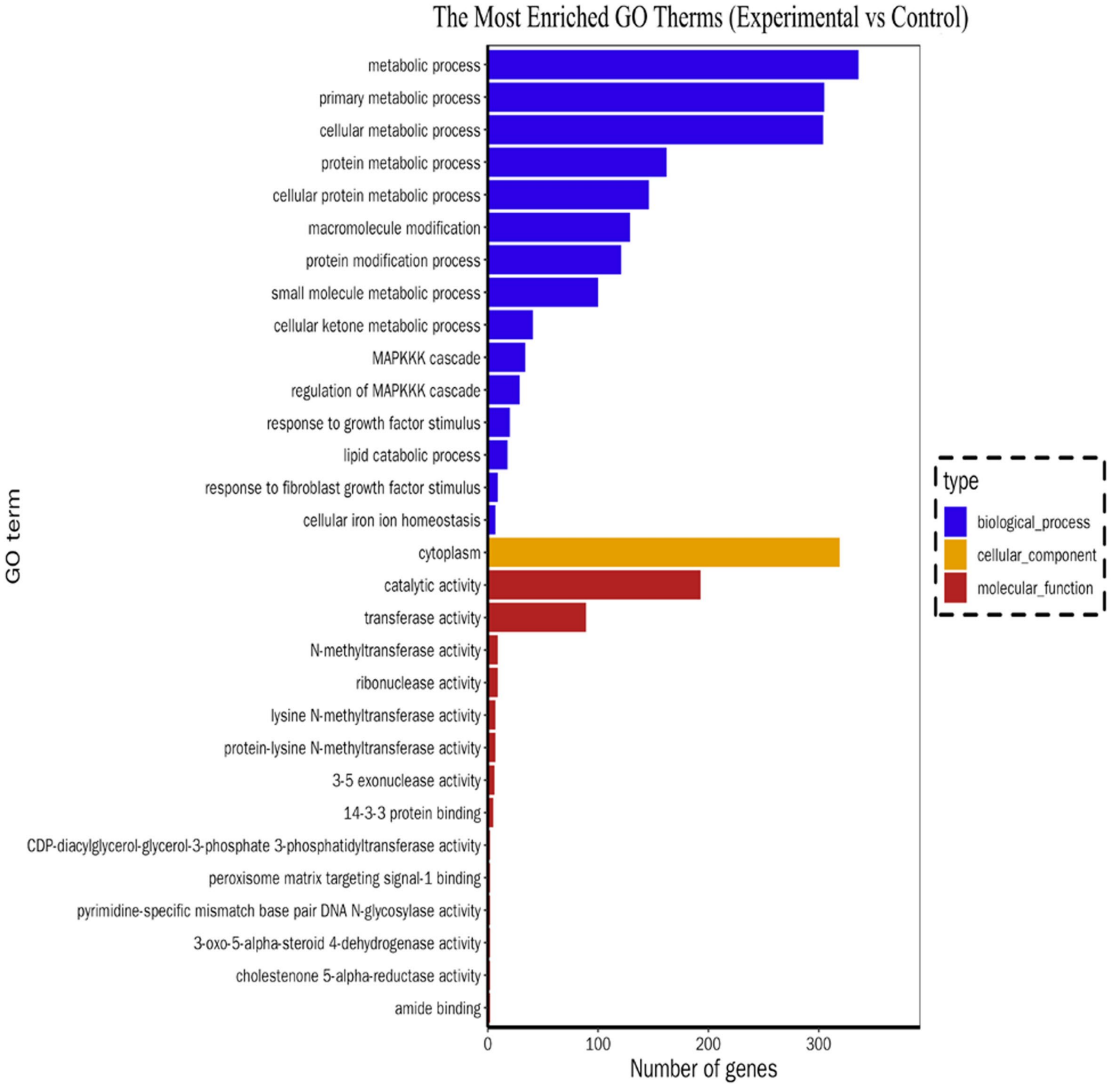
Histogram of GO enrichment of DE miRNAs.

**Figure 8:**
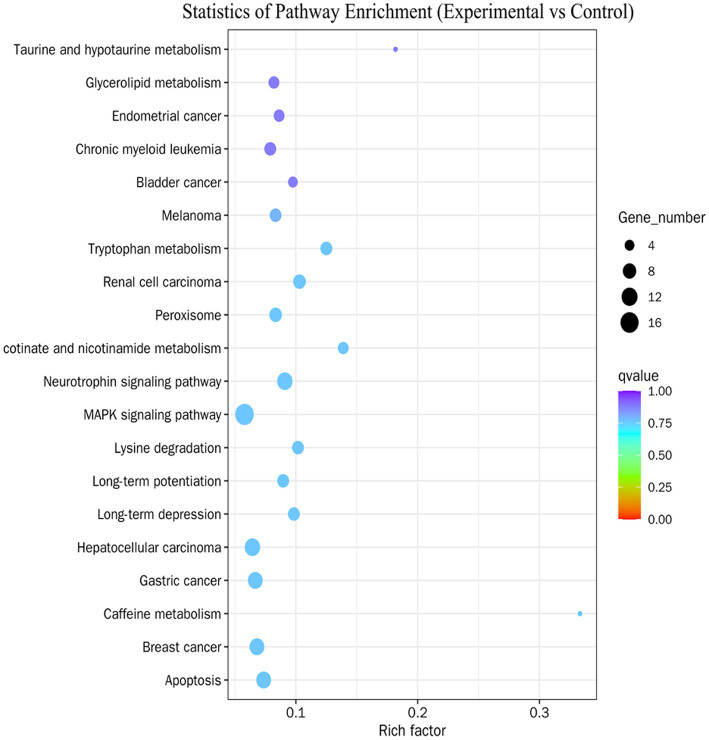
KEGG enrichment of DE miRNA. The rich factor on the abscissa indicates the number of DE miRNA located in the KEGG. Pathways are shown on the ordinate.

GO and KEGG enrichment analyses were used to examine the functions of DE miRNAs in infected and uninfected RAW 264.7 cells. GO annotation analysis of the DE miRNAs showed that DE miRNA in the three GO categories of biological process, cellular component, and molecular function. The GO terms with the most significant enrichment were used to create the GO functional annotation map (Figure 7). KEGG enrichment analysis revealed that the DE miRNAs were collectively enriched in 275 signaling pathways, which primarily involve various biochemical, metabolic, and signal transduction processes within cells. Based on enrichment significance (Q-value), representative pathways were selected, including apoptosis, MAPK signaling pathway, tryptophan metabolism, and lysine degradation (Figure 8). Further analysis indicated that a substantial number of upregulated miRNAs (such as *miR-3473b*, *miR-1938*, *miR-6963-5p*, etc.) were enriched in the MAPK signaling pathway and apoptosis pathway, with their targeted mRNAs widely participating in processes such as inflammatory responses, cytokine production, oxidative stress, antimicrobial responses, and apoptosis. These findings suggest that DE miRNAs may participate in the host’s early response to *Salmonella* by regulating the aforementioned pathways. In addition, metabolic pathways such as tryptophan metabolism and lysine degradation were also significantly enriched, with some DE miRNAs (such as *miR-150-5p*) predicted to target metabolism-related genes, indicating that DE miRNAs may influence immune function by modulating metabolic reprogramming in macrophages.

### 3.6 Construction of miRNA-mRNA regulatory network

**Figure 9:**
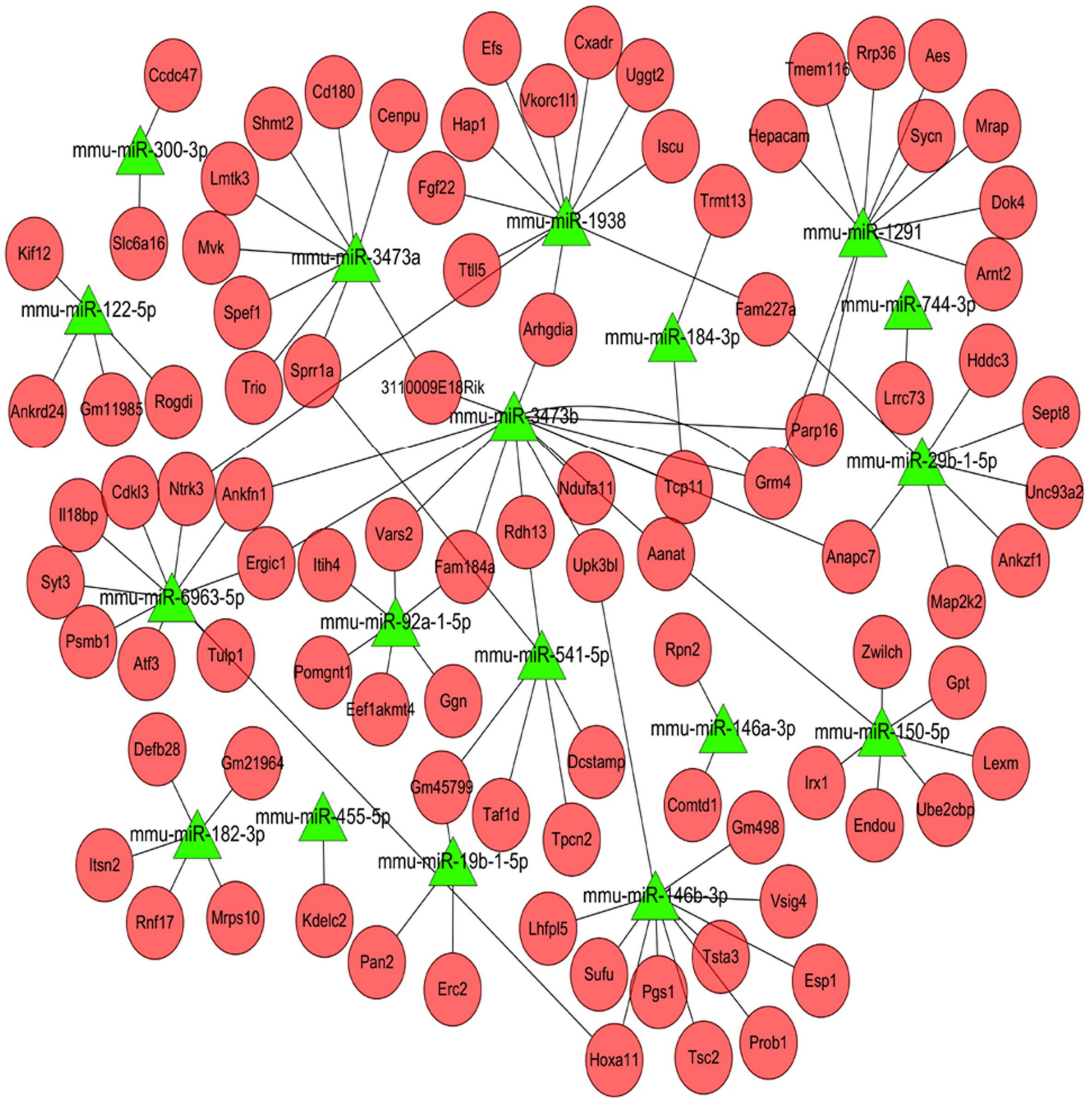
MiRNA-mRNA correlation network. Green triangles represent miRNAs and red circles represent mRNAs.

The targets of DE miRNAs in mRNAs were predicted by miRanda software. The results showed that 18 DE miRNAs were predicted to target 93 DE mRNAs. Based on these interactions, a regulatory network was constructed and visualized using Cytoscape (Figure 9). The analysis revealed that *miR-3473b*, *miR-146b-3p*, and *miR-1938* showed the highest number of predicted target mRNAs and shared commonly targeted mRNAs, suggesting that these DE miRNAs may play key roles in regulating transcriptional in response to infection. Furthermore, comparison of the expression levels of the DE miRNAs and their targeted DE mRNAs showed that the expression of some DE miRNAs and their corresponding DE mRNAs was negatively correlated, further indicating the potential existence of negative regulation at the post-transcriptional level (Table 5).

**Table 5:** DE miRNAs and DE mRNAs with a negative regulatory relationship.

DE miRNA		Target DE mRNA	
Gene name	Log2 (fold change)	Gene name	Log2 (fold change)
*miR-1291*	1.0526	*Hepacam*	−1.6875
*miR-1938*	1.2931	*Hap1*	−1.6158
*miR-3473a*	1.0822	*Spef1*	−1.9091
*miR-184-3p*	1.4513	*Tcp11*	−2.54
*miR-3473b*	1.2492		
		*Ctsk*	−1.1677
		*Osbpl5*	−1.3043
		*Pias3*	−1.2219
		*Fgfr4*	−3.6778
		*Map3k3*	−1.0788
		*Nbr1*	−1.7369
		*Pnck*	−1.6875
*miR-455-5p*	1.2173	*Kdelc2*	−1.3308
*miR-6963-5p*	1.4156	*C530008M17Rik*	−1.0634

### 3.7 DE mRNA and DE miRNA RT-qPCR verification

**Figure 10:**
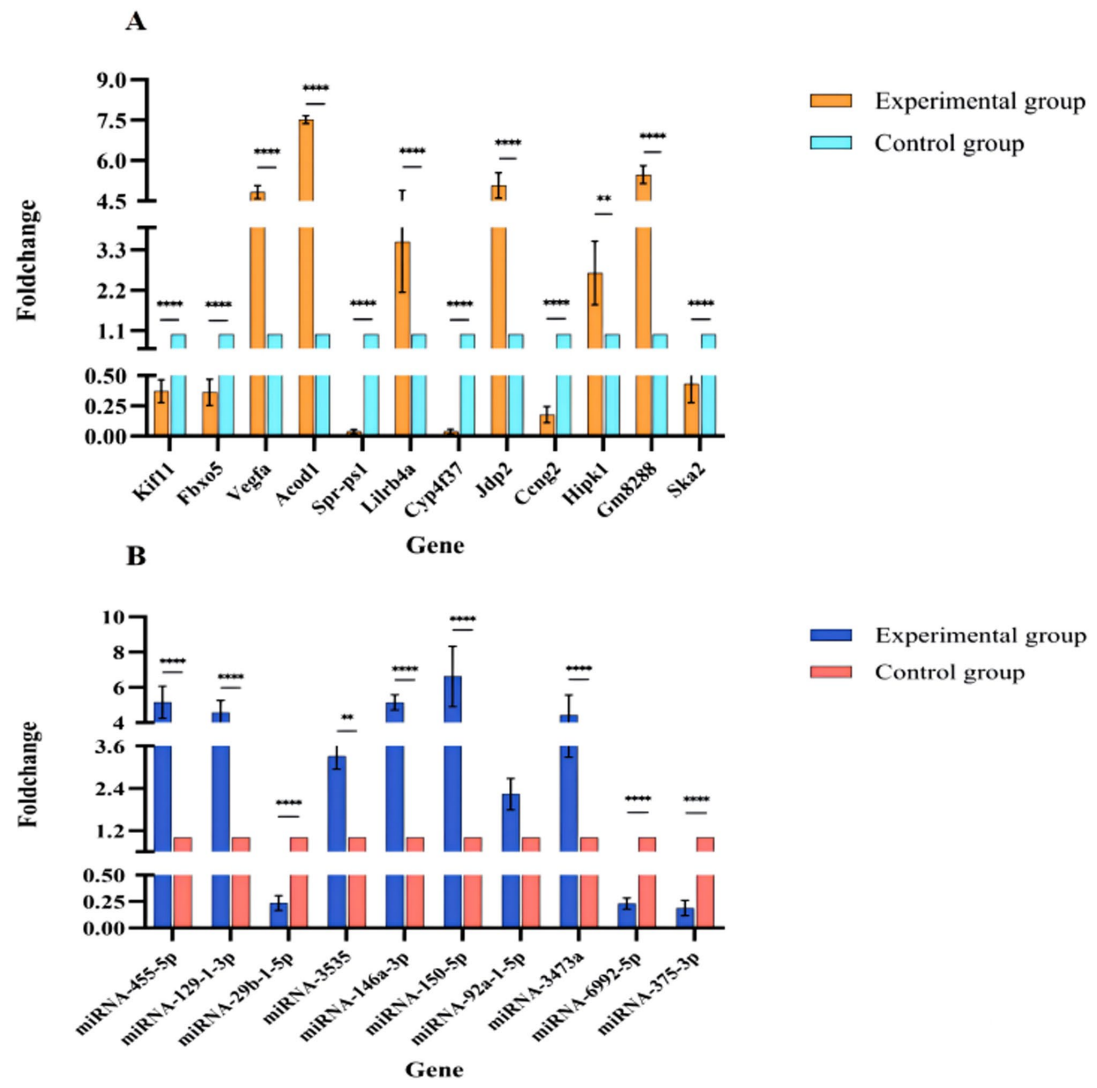
**(A)** RT-qPCR verification. Expression of selected DE mRNAs verified with RT-qPCR. Genes are shown on the abscissa and fold-change values on the ordinate. **(B)** RT-qPCR verification. Expression of selected DE miRNAs verified with RT-qPCR. miRNAs are shown on the abscissa and fold-change values on the ordinate.

To verify the accuracy of the sequencing results, a total of 12 mRNAs (*Acod1*, *Gm8288*, *Jdp2*, *Vegfa*, *Lilrb4a*, *Hipk1*, *Ska2*, *Fbxo5*, *Kif11*, *Ccng2*, *Spr-ps1*, and *Cyp4f37*) and 10 miRNAs (*miR-146a-3p*, *miR-3473a*, *miR-3535*, *miR-455-5p*, *miR-150-5p*, *miR-92a-1-5p*, *miR-129-1-3p*, *miR-375-3p*, *miR-6992-5p* and *miR-29b-1-5p*) were randomly selected for RT-qPCR verification, with three biological replicates performed for each gene. The results were found to be consistent with the sequencing data, indicating the reliability of the results (Figures 10A,B).

## 4 Discussion

*Salmonella* is a common pathogen that infects both humans and animals, causing a variety of diseases. To date, over 2000 *Salmonella* serotypes have been reported worldwide. These show significant diversity and can threaten both human health and the livestock breeding industry (35). Among many serotypes of *Salmonella*, *Salmonella* Dublin is defined as host-adapted and can cause enteritis and/or systemic disease in animals of different ages. Although *Salmonella* Dublin is adapted to cattle, natural infections with *Salmonella* Dublin may also occur in other animal species, in particular small ruminants, and the pathogen is considered a public health concern worldwide due to its high virulence and association with systemic disease (36).

In recent years, the application of high-throughput sequencing has greatly enhanced the ability to understand interactions between host macrophages and *Salmonella* pathogens (37–39). Macrophages are an important component of the innate immune system associated with non-specific immunity in animals, and participate in key immune responses such as antigen presentation, pathogen phagocytosis, inflammation, and the maintenance of tissue homeostasis (40, 41). To date, most studies on the inflammatory response have focused primarily on mRNAs, although miRNAs have been found to be important in regulating mRNA expression (34, 42). Macrophages play an important role in host-pathogen interactions associated with salmonellosis, and they can become a safe haven for the survival and spread of *Salmonella*. Advances in high-throughput transcriptome sequencing and extensive database resources provide a strong foundation for the understanding of gene expression in macrophages in response to bacterial infection (43). In this study, transcriptome sequencing technology was used to analyze the transcriptome of RAW264.7 cells infected with *Salmonella*, providing a theoretical basis for elucidating the immune-related functions of macrophages during the early stage of *Salmonella* infection.

After infecting macrophages, *Salmonella* can cativate the NF-κB and the IFN-*γ* pathways in the cell through effector proteins secreted by its type III secretion system. This induces M1-type polarization of the macrophages,leading to increased transcription of pro-inflammatory factors, such as TNF-*α*, IL-1β, and IL-6, and triggering an inflammatory response (44). In this study, sequencing of the mRNA transcriptome revealed significant changes in the expression of multiple mRNAs in macrophages following infection with *Salmonella* Dublin. Among these, the most substantially upregulated were the immunometabolism-related gene *Acod1* and the chemokine-related gene *Ccl22*. KEGG enrichment analysis indicated that the DE mRNAs were primarily involved in pathways associated with metabolic responses, apoptosis, and signal transduction. Specifically, *Acod1* and *Ccl22* were enriched in the metabolic pathway and the chemokine signaling pathway, respectively. Furthermore, marked upregulation of the anti-apoptotic gene *Bcl2a1b* was observed in apoptosis-related pathways. These results suggest that *Acod1*, *Ccl22*, and *Bcl2a1b* may play key roles in the macrophage response to *Salmonella* Dublin infection.

Current research indicates that the expression of *Acod1*, a key regulator of immunometabolism, can be induced by various inflammatory mediators. The protein it encodes catalyzes the conversion of cis-aconitate to itaconate and promotes the production of mitochondrial reactive oxygen species (mROS), thereby linking immune and metabolic processes and enhancing antibacterial activity (45–47). Apart from its participation in metabolic pathways leading to the production of antibacterial factors such as itaconate, *Acod1* is also associated with various aspects of the immune response, such as, for example, mediating the upregulation of TNF-*α*-induced protein 3 to regulate NF-κB signaling. In addition, *Acod1* can modulate Toll-like receptor signaling and the expression of interferon-stimulated genes, thereby modulating inflammatory responses (48–53). Based on these findings as well as those of the present study, it is hypothesized that during the early stage of *Salmonella* Dublin infection, upregulation of *Acod1* enhances the ability of the macrophage to resist the pathogen via the activation of metabolic pathways that lead to the production of itaconate and mROS.

As *Salmonella* infection can induce M1-type macrophage polarization and mediate inflammatory responses, it is likely that persistent M1 polarization and excessive inflammation may lead to cellular damage. Therefore, macrophages often shift toward an M2-type polarization state to balance the immune response. This study observed significant upregulation of *Ccl22* expression in macrophages after infection with *Salmonella* Dublin. CCL22 is an important chemokine that can induce M2 polarization in macrophages, suppress excessive inflammatory responses, and promote immune homeostasis. Moreover, CCL22 can bind to the CCR4 receptor to recruit immunomodulatory cells such as regulatory T cells, thereby indirectly promoting macrophage activation and triggering the immune response (54). The results of this study suggest that during the early stage of *Salmonella* Dublin infection, the CCL22 chemokine drives the transition of macrophages toward M2-type polarization while activating chemokine signaling, recruiting immunomodulatory cells, and inducing immune responses in macrophages, thereby maintaining the homeostasis of the immune response.

Studies have shown that *Salmonella* can mitigate inflammatory responses and evade immune surveillance by inducing apoptosis (55, 56). The present study observed significant upregulation of *Bcl2a1b*, which encodes an anti-apoptotic protein that plays a crucial role in cellular anti-apoptotic regulation, particularly in the context of mitochondrial apoptosis. Research by Shi J et al. indicates that Bcl2A1B interacts with pro-apoptotic proteins and cross-regulates NF-κB signaling and autophagy, making it essential for cell survival and stress responses (57). Based on the above results, it is speculated that macrophages can mitigate apoptosis induced by early-stage *Salmonella* Dublin infection, enabling their survival and continued immune function by upregulation of *Bcl2a1b*, thereby preventing apoptosis.

At the miRNA level, this study observed that macrophage expression of multiple miRNAs was significantly altered after *Salmonella* Dublin infection, with notable upregulation of *miR-146a-3p*, and *miR-150-5p*, among others. KEGG analysis indicated that the DE miRNAs were primarily enriched in pathways related to signal transduction, metabolism, and apoptosis. It has been reported that *miR-146a-3p* functions as a negative regulator by targeting key molecules in the TLR/NF-κB pathway to suppress excessive inflammatory responses (58, 59). The significant upregulation of *miR-146a-3p* observed in this study suggests that during the early stages of *Salmonella* Dublin infection of macrophages, a negative regulatory mechanism may be initiated to prevent sustained activation of inflammatory signaling. This mechanism may work synergistically with CCL22-mediated M2 polarization to regulate the immune response. Furthermore, KEGG enrichment revealed that *miR-146a-3p* is also involved in metabolic regulation, and its potential targets may indirectly affect the activation of ACOD1-related metabolic pathways, thereby influencing immunometabolic responses. Furthermore, this study observed widespread enrichment of *miR-150-5p* in metabolic pathways. It has been reported that overexpression of *miR-150-5p* can alleviate LPS-induced inflammatory responses and apoptosis in RAW264.7 cells (60). Based on these findings, it is speculated that *miR-150-5p* may also participate in immunometabolic responses and function synergistically with Bcl2A1B to mitigate apoptosis, collectively ensuring macrophage survival and immune function.

Integrating the above results, this study suggests that after *Salmonella* Dublin infection, macrophages may enhance host defense through the various synergistic mechanisms. On one hand, upregulating the expression of *Acod1* and *miR-146-3p* can activate metabolic pathways, leading to the production of itaconate and mROS to enhance antibacterial activity. On the other hand, CCL22-mediated promotion of M2 polarization modulates the balance of the immune response, with recruitment of immune regulatory cells for further coordination of the response. Simultaneously, the increased levels of Bcl2A1B and *miR-150-5p* inhibit apoptosis, thereby maintaining macrophage survival and immune function. The synergistic action of these three mechanisms, enables macrophages to mount a defense against *Salmonella* infection. This study, despite the identification of multiple DEGs and analysis of some of their functions, nevertheless has several limitations. For example, the study used mouse RAW 264.7 cells, rather than bovine monocyte macrophages. However, RAW 264.7 cells, as a classic model for macrophage biology research, have been widely employed in studies investigating the interaction between bovine pathogens and the host, demonstrating a high degree of consistency with bovine primary macrophages in terms of key immune signaling pathways and functional responses (61, 62). This model has accumulated considerable comparable data in previous studies, providing a reliable *in vitro* platform for the rapid screening of key molecular events during the early stage of *Salmonella* Dublin infection in this study. Furthermore, cells were only analyzed at the early stage of infection (2 h), without the inclusion of further time points. In future research, we will conduct more studies on bovine monocyte macrophages infected with *Salmonella* Dublin, thereby providing specific insight into pathogen interactions with the defense strategies of host cells.

## 5 Conclusion

This study identified 1,080 DE mRNAs and 23 DE miRNAs, and suggests that macrophages may resist early *Salmonella* Dublin infection through three synergistic mechanisms. The first step involves upregulation of the expression of *Acod1* and *miR-146-3p*, leading to the activation of metabolic pathways and the production of itaconate and mROS, thereby enhancing antibacterial activity. The second step utilizes CCL22 to drive macrophage polarization toward the M2 phenotype, enabling maintenance of immune homeostasis and the recruitment of additional immunomodulatory cells for a coordinated defense. Finally, Bcl2A1B and *miR-150-5p* are upregulated to inhibit apoptosis, a strategic approach that maintains macrophage survival and preserves their immune function during infection.

## Data Availability

The data used in this article are provided by the authors without reservation. The raw data from the mRNA and miRNA sequencing were submitted to the NCBI, with accession numbers PRJNA1270173 (https://www.ncbi.nlm.nih.gov/bioproject/PRJNA1270173/) and PRJNA1272797 (https://www.ncbi.nlm.nih.gov/bioproject/?term=PRJNA1272797), for the mRNA and miRNA data of the *Salmonella* Dublin-infected and control groups, respectively. The data associated with the figures in this study have been archived in the Figshare database and are accessible via the DOI: 10.6084/m9.figshare.30963655.

## Footnotes

1
http://www.cytoscape.org/
