# Supplementary material for: Analysis of mRNA and miRNA expression in RAW 264.7 macrophages infected with Salmonella enterica subsp. enterica serovar Dublin in the early stage of infection
Source: Front Vet Sci. 2026 May 4;13:1794290. doi: 10.3389/fvets.2026.1794290 (PMC13180857; doi:10.3389/fvets.2026.1794290)
Supplement: Supplementary file 1 [file Table_1.DOCX]

Table S1 Validation of gene primer sequences

| Primer name | Primer sequence (5´→3´) | Annealing temperature (℃) | Amplification efficiency (%) |
| --- | --- | --- | --- |
| *β*-actin-F | TTCCGCCTTAATACTTCATT | 60 | 98.2 |
| *β*-actin-R | GACCAAAGCCTTCATACATC |  | 98.2 |
| U6-F | CTCGCTTCGGCAGCACA | 60 | 96.5 |
| U6-R | AACGCTTCACGAATTTGCGT |  | 96.5 |
| *Acod1*-F | ATGGTATCATTCGGAGGAG | 60 | 101.3 |
| *Acod1*-R | TATTGGGTGACTTTATGGA |  | 101.3 |
| *Gm8288*-F | CTCCAAGAAGTGCCCAGAA | 60 | 97.8 |
| *Gm8288*-R | CTTTCATAATGAGCCCTGT |  | 97.8 |
| *Jdp2*-F | CAACCCGTGAAGAGTGAGC | 60 | 99.4 |
| *Jdp2*-R | TTCTTGTTCCGGCATCTGG |  | 99.4 |
| *Vegfa*-F | TGTCACCACCACGCCATCA | 60 | 102.1 |
| *Vegfa*-R | CGGACCCAAAGTGCTCCTC |  | 102.1 |
| *Lilrb4a*-F | ATTCAGATGCTATGGCTAC | 60 | 95.6 |
| *Lilrb4a*-R | CTAAGTGATTATGGGTGGA |  | 95.6 |
| *Hipk1*-F | GCACCTACTACATTCAAACCT | 60 | 98.9 |
| *Hipk1*-R | GAAAGTCAAACAAGCCCTC |  | 98.9 |
| *Ska2*-F | GCCTGACATTTCCCTTGAC | 60 | 97.2 |
| *Ska2*-R | TTATTTCCATCCCATCCTT |  | 97.2 |
| *Fbxo5*-F | CTCCAGTCAGCGTGGTCAG | 60 | 100.8 |
| *Fbxo5*-R | GAGGCTTTCGTTGTTCTTC |  | 100.8 |
| *Kif11*-F | ACCAGAGCACCTACCTAAA | 60 | 96.1 |
| *Kif11*-R | TAGTGAGACTGTGCCAAAA |  | 96.1 |
| *Ccng2*-F | CGCATCAGTCAGTGTAAAT | 60 | 97.5 |
| *Ccng2*-R | GGTAGCTTCCAGCTCATAG |  | 97.5 |
| *Spr-ps1*-F | GATAAAGACAAACCAACCCTG | 60 | 99 |
| *Spr-ps1*-R | CTTGAACGCCACTCCCATA |  | 99 |
| *Cyp4f37*-F | TGGACGCTGTGACTCAACC | 60 | 100.2 |
| *Cyp4f37*-R | TGCCAGGACTTTCTATGTATT |  | 100.2 |
| *miRNA*-R | AGTGCAGGGTCCGAGGTATT | - | - |
| *miRNA-146a-3p*-F | CGCGCCTGTGAAATTCAGTT | 60 | 94.7 |
| *miRNA-3473a*-F | GCGCGTGGAGAGATGGC | 60 | 96.3 |
| *miRNA-3535*-F | CGCGTGGATATGATGACTGATTAC | 60 | 92.8 |
| *miRNA-455-5p*-F | CGCGTATGTGCCTTTGGACT | 60 | 98.1 |
| *miRNA-150-5p*-F | GCGTCTCCCAACCCTTGTA | 60 | 95.4 |
| *miRNA-92a-1-5p*-F | CGAGGTTGGGATTTGTCGC | 60 | 97.6 |
| *miRNA-129-1-3p*-F | CGAAGCCCTTACCCCAAA | 60 | 96.9 |
| *miRNA-375-3p*-F | GCGTTTGTTCGTTCGGCTC | 60 | 99.2 |
| *miRNA-6992-5p*-F | CGTGCCTGTGATGGTTTGG | 60 | 93.5 |
| *miRNA-29b-1-5p*-F | GCGGCTGGTTTCATATGGT | 60 | 97 |
